# Anesthetic management of a patient with limb-girdle muscular dystrophy 2B:CARE-compliant case report and literature review

**DOI:** 10.1186/s12871-019-0813-8

**Published:** 2019-08-17

**Authors:** X. Q. Cao, K. Joypaul, F. Cao, L. L. Gui, J. T. Hu, W. Mei

**Affiliations:** 1 Department of Anesthesiology and Pain Medicine, Tongji Hospital, Tongji Medical College, Huazhong University of Science and Technology, 1095 Jiefang Road, Wuhan, 430030 People’s Republic of China; 2 Department of Anesthesiology, Flacq Hospital, Hospital Road, Centre-de-Flacq, Mauritius; 3 Department of Psychiatry, University of Missouri-Kansas City, 5100 Rockhill Road, Kansas City, 64110 MO USA; 4 Department of Anesthesiology, Wuhan No.4 Hospital, 473 Hanzheng Road, Wuhan, 430033 People’s Republic of China

**Keywords:** Limb-girdle muscular dystrophy 2B, Anesthesia, Surgery

## Abstract

**Background:**

Limb-girdle muscular dystrophies (LGMDs) belong to few neuromuscular disorders mainly involving pelvic and shoulder girdle muscles. Also, cardiac or pulmonary complications, increased rhabdomyolysis risk when exposed to volatile anesthetics and succinylcholine may increase anesthesia related risks. However, current reports about the anesthesia management of these patients are limited.

**Case presentation:**

We described our anesthetic management of a 36 years old woman with LGMD 2B receiving arthroscopic knee surgery. In consideration of the high risk of rhabdomyolysis, total intravenous anesthesia (TIVA) was selected for her surgery. Considering the unpredictable respiratory depression, opioid based patient-controlled intravenous analgesia was replaced with an intra-articular cocktail therapy consisting of 20 ml of 0.2% ropivacaine. Also, we reviewed the literatures on anesthetic management of LGMD through searching PubMed, in order to provide a comprehensive and safe guidance for the surgery.

**Conclusions:**

Carefully conducted general anesthesia with TIVA technique is a good choice for LGMD patients. Neuraxial anesthesia may be used if general anesthesia needs to be avoided. To warrant safe anesthesia for surgery, any decision must be well thought out during perioperative period.

## Background

Limb-girdle muscular dystrophies (LGMDs) belong to few very rare neuromuscular disorders mainly involving pelvic and shoulder girdle muscles [1]. The estimated incidence of this disease is 1 to 6.5 in 100,000 [2]. Although named as the typical muscle, LGMDs are actually a group of systemic diseases and may be complicated with cardiac or pulmonary involvements [3]. Increased risk of rhabdomyolysis and malignant hyperthermia (MH) when exposed to volatile anesthetics and succinylcholine are life-threatening complications [1]. However, the current reports about anesthetic management of LGMD patients are limited. We aimed to present our clinic experience about a patient with LGMD 2B receiving arthroscopic knee surgery. Then, we reviewed literatures on anesthetic management of LGMD published in PubMed, in order to provide a comprehensive and safe guidance for surgery. The reporting of this case was approved by the Research and Ethics Committee of Tongji Hospital at Tongji Medical College, Huazhong University of Science and Technology (TJ-IRB20180902). The patient had gave written informed consent for reporting and publishing her clinical data and images.

## Case presentation

**Fig. 1:**
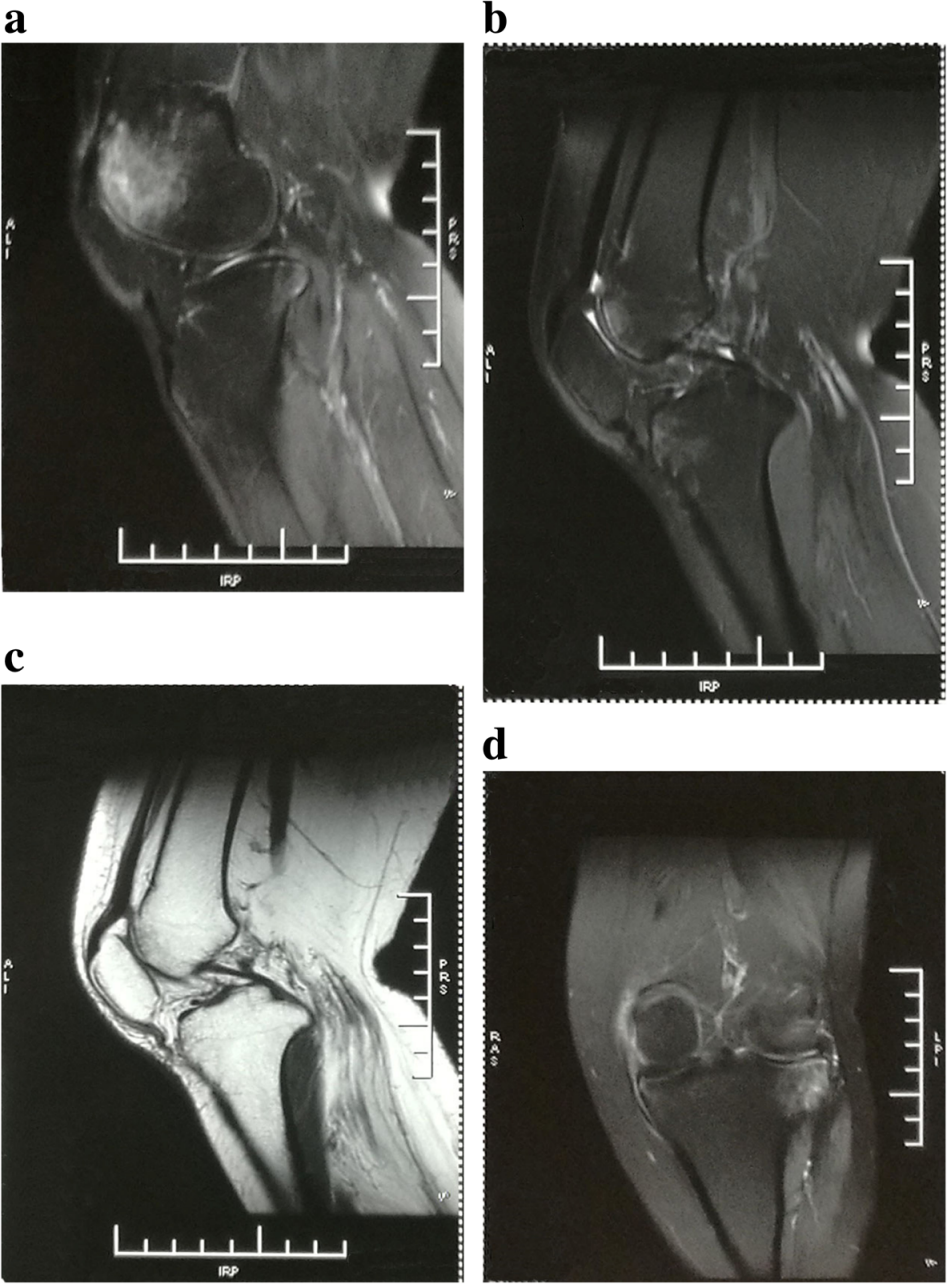
Left knee joint magnetic resonance imaging. It revealed left knee joint injury. **a** Osteoedema of distal femur; **b** Rupture of anterior cruciate ligament; **c** Relaxation of quadriceps femoris tendon and patellar ligament; **d** Posterolateral osteoedema of tibial plateau

A 36 years old woman, 54 kg in weight, 1.60 m tall, BMI 21.09, was hospitalized with left knee joint injury and was scheduled for arthroscopy of left knee (Fig. 1). She had a history of LGMD type 2B diagnosed in 2013 by muscle biopsy. Genetic testing showed homozygous DYSF mutation on 11 and 33 exons, and MRI revealed fat infiltration and fluid collection within the lower limb muscles. Her symptoms predominantly affected her lower limbs. Her initial symptom presented as difficulty in climbing stairs at 24 years old. During her admission, she did not have any muscle weakness in the upper limbs, dysphagia, or other clinical manifestations. Her symptoms were slow in progression, with inability to climb the stairs by 2011. She underwent multiple examinations for muscle enzyme levels, electromyography (EMG), etc., and she was also once misdiagnosed as ‘polymyositis’. Aspartate aminotransferase (AST), alanine aminotransferase (ALT) and creatine kinase (CK) levels were high. She underwent an uneventful cesarean section under spinal anesthesia in February of 2017. Her younger brother was also diagnosed with LGMD type 2B.

**Fig. 2:**
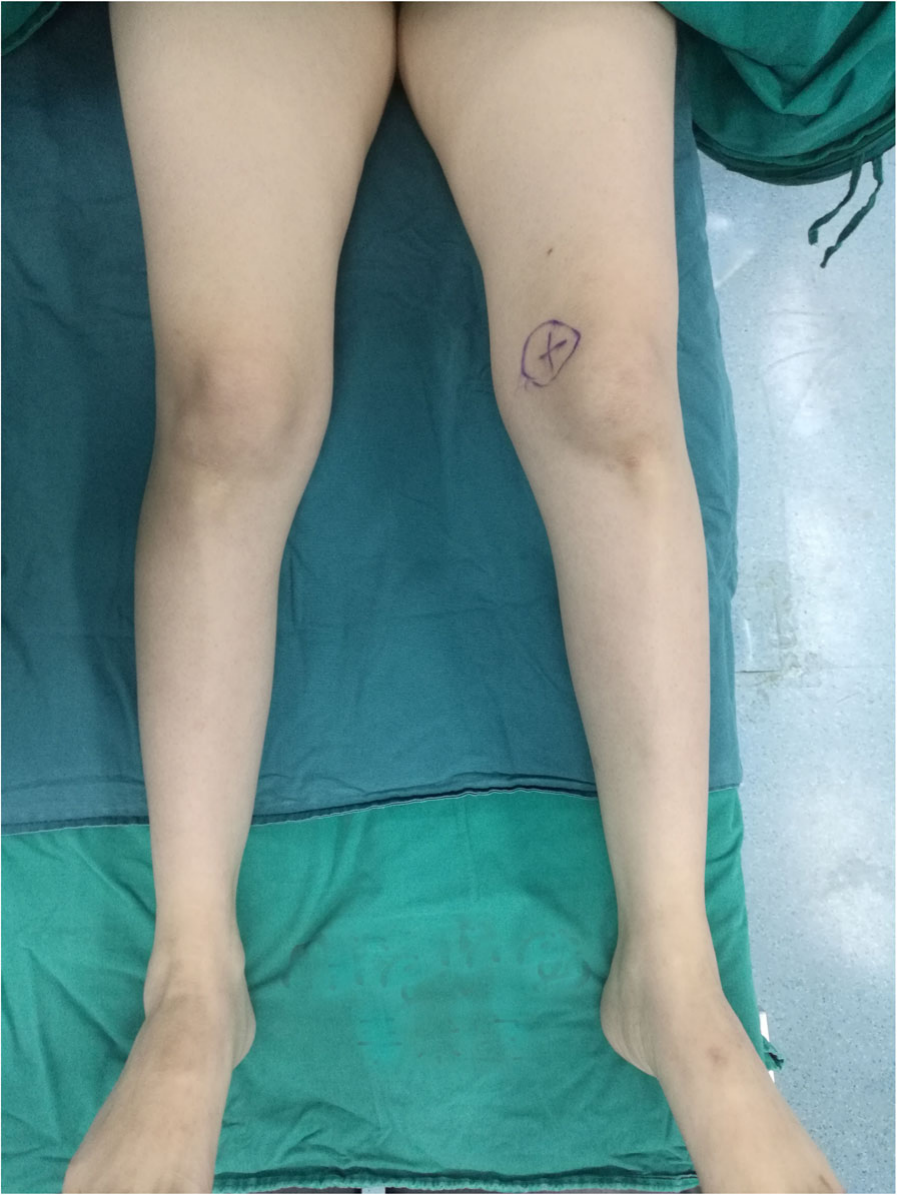
photograph of patient’s lower limbs. It showed muscles of lower limbs atrophy slightly. Muscle strength of the lower limbs was at level 4, whereas muscle tension was decreased

Her current physical examination was remarkable, muscle strength, muscle tension, and muscular volume of the upper limbs were all found to be normal. Muscle strength of the lower limbs was at level 4, whereas muscle tension was decreased (Fig. 2). MRI of lower limbs revealed atrophy of quadriceps femoris, adductor muscles, semimembranosus, and hypertrophy of gracilis as well as the long head of biceps femoris. Her complete blood count, blood chemistry and urinalysis were unremarkable. Abnormal laboratory findings were listed as follows: aspartate aminotransferase 83 U/L, alanine transaminase 43 U/L and creatine kinase 3479 U/L. The electrocardiogram (ECG) showed T wave anomaly in the anterior wall. Chest X-ray revealed increased lung markings. After entering the operating room, the patient underwent transthoracic echocardiography and revealed good left ventricular functions and no evidence of cardiomyopathy or pulmonary hypertension.

The patient received adequate explanation about the increased risk of anesthesia and gave her informed consent. In consideration of the high risk of rhabdomyolysis and malignant hyperthermia, we decided to proceed with total intravenous anesthesia (TIVA) for her elective surgery.

Before the patient was transferred to operating room, the anesthesia apparatus was substituted by a respirator connected with a new breath circuit for necessary isolation from volatile anesthetics. After the patient arrived in the operation room, electrocardiogram and pulse oximetry saturation (SpO_2_) monitoring was immediately started. Heart rate was 74 bpm, SpO_2_ was 98% in room air. Noninvasive blood pressure (NIBP) was 113/62 mmHg. To avoid the potential muscular damage caused by repeated cuff pressure measurements, invasive blood pressure monitoring was used. Except for the standard monitoring, end-tidal CO_2_ (EtCO_2_) and nasopharyngeal temperature monitoring were also important. Flurbiprofen 50 mg was given intravenously as preventive analgesia regime. Anesthesia induction was performed with 50 mg propofol and 20 mg etomidate after 30μg sufentanil, a size 3# laryngeal mask airway was inserted 2 min later to secure the airway. Muscle relaxant was not used to avoid the delayed recovery of muscular strength. Propofol (6-12 mg.kg^− 1^.h^− 1^) and remifentanil (0.1–0.2 μg.kg^− 1^.min^− 1^) were pumped to maintain anesthesia. Non-tourniquet technology was adopted during the operation to avoid potential muscle damage caused by tourniquet, and controlled hypotension was used to reduce the risk of bleeding. During the operation, heart beat was between 55 and 82 bpm, while the mean blood pressure remained between 70 and 83 mmHg. EtCO_2_ was maintained at around 40 mmHg. Nasopharyngeal temperature was monitored at 36.2–36.4 °C. After the surgical intervention which lasted around 1 h and 48 min, the patient recovered rapidly approximately within 2 min after cessation of TIVA. Considering the unpredictable respiratory depression, opioid based patient-controlled intravenous analgesia was not used. Instead an intra-articular cocktail therapy consisting of 20 ml of 0.2% ropivacaine was injected at the end of surgery acting as a substitute for postoperative analgesia.

During the follow-up on the second postoperative day, the resting visual analog scale (VAS) pain score was 2 and the VAS pain score at movement was 4. The patient could be mobilized out-of-bed, didn’t complain of any significant discomfort during movement of the lower limbs. She was discharged on the fifth day after surgery.

Then, we reviewed relevant literatures published in PubMed as of September 2018. The search was limited to studies about humanity. Only English cases were included. The keywords used for the search included [“limb-girdle muscular dystrophy” OR “limb-girdle muscular dystrophy” OR “LGMD”] and [“anesthetic management” OR “anesthesia” OR “anaesthetic management” OR “anaesthesia”].

## Discussion and conclusion

LGMD constitutes a group of genetic disorders originally devided into autosomal dominant (LGMD type 1) and autosomal recessive (LGMD type 2) [4–6], 8 subtypes of LGMD1 and 26 subtypes of LGMD2 are currently recognized [3]. Successive letters of the alphabet have been used to name the LGMDs according to the identification chronology of genetic locus. The pathogenesis of LGMD is the mutation of causative genes leading to abnormal protein synthesis localized to various parts of the muscle fiber [2]. Degeneration and necrosis of muscle fibers, which are replaced by connective tissue and fat is the classic pathologic change [7]. However, the biopsy findings among the various LGMDs lack in specificity and significant overlap. Also, Serum creatine kinase (CK) levels couldn’t reflect the severity of LGMD [3]. Genetic testing is necessary for diagnosis of LGMD.

The mutations of dysferlin gene (DYSF, MIM*603009) located on chromosome 2p13.3 induces abnormal dysferlin protein synthesis is the reason of LGMD 2B [8]. The symptom onset ranges from adolescence to late adulthood (17–30 years of age), the disease progresses fairly slowly, and approximately 10% of patients may be confined to a wheelchair [9]. Serum CK levels are considerably high, usually up to 40 times of the normal [10, 11].

The use of inhalation anesthetics and succinylcholine may lead to threatening hyperkalemia and rhabdomyolysis, and should be avoided [12–14]. Propofol, etomidate and opioids are proved to be trigger-free agents [15, 16]. However, myopathic patients are more sensitive to anesthetics, opioids and muscle relaxants [17]. In order to minimize the risk of postoperative respiratory depression, short acing anesthetic agents are the optimum selection [14]. Propofol, as it’s short-acting, easiness in titration, is suitable for LGMD [1]. Remifentanil was also unlikely to cause postoperative respiratory failure and prolonged sedation. Therefore, we chose total intravenous anesthesia (TIVA) by infusing propofol and remifentanil for anesthetic maintenance of this patient. Sufentanil 30μg was also used in this patient to provide adequate analgesia. Considering the high sensitivity and prolonged duration of action to muscle relaxants in these patients, muscle relaxants were avoided in our case. Therefore, for this patient laryngeal mask airway is more beneficial as compared to an endotracheal intubation. Intravenous patient-controlled analgesia (iv-PCA) based on opioids may lead to unpredictable respiratory depression, and was not considered in our case. Instead, intra-articular injection of cocktail therapy with 0.2% ropivacaine 20 ml had been used with an excellent outcome.

Previous reports were searched through PubMed as of September 2018 using the following key words: [“limb-girdle muscular dystrophy” OR “limb girdle muscular dystrophy” OR “LGMD”] and [“anesthetic management” OR “anesthesia” OR “anaesthetic management” OR “anaesthesia”]. Only English reports were included. Six cases were reviewed. Time of publication, LGMD type, symptoms, complications, operative procedures, preoperative examinations, intraoperative monitoring, anesthesia method, anesthesia machine preparation, anesthesia induction, anesthesia maintenance, pain management after operation are summarized in Table 1.

**Table 1:** literature review of anesthetic management of patients with LGMD

Title	Author/year	Age/gender	LGMD type	Complication	Operative procedure	Pre-operational examinations	Anesthesia method	Anesthesia machine preparing	Anesthesia induction	Anesthesia maintenance	Intraoperativemonitoring	Pain management after surgery
Anaesthetic management of a patient with limb-girdle muscular dystrophy for laparoscopic cholecystectomy [18]	Freda C. Richa/2010	A 57-year-old man	LGMD1	severe restrictive lung disease	laparoscopic cholecystectomy/45 min	ECG, Chest X-ray,echocardiography	General anaesthesia (TIVA)	A disposable circuit, fresh CO2-absorbent, disconnecting the vaporisers, flushing with O2 at a rate of 10 L. min^− 1^ for 20 min	1 mg.kg^− 1^ .min^− 1^remifentanil infusing for 1 min, 3 mg. kg^− 1^ propofol iv	TIVA by using 0.1–0.4 mg.kg^− 1^ min^− 1^ remifentanil and 6–9 mg. kg^− 1^ .h^− 1^ Propofol infusion	ECG, pulse oximetry, end-tidal CO_2_, invasive arterial pressure, rectal temperature, BIS	40 ml 0.125% bupivacaine aerosolised intra-peritoneally and 1 g paracetamol given intravenously
Anaesthetic Management of a Child with Limb-Girdle Muscular Dystrophy [19]	Gamze Sarkılar/2014	An 8-year old boy	–	–	Appendectomy/16 min	–	General anaesthesia (TIVA)	a disposable patient circuit, flushing soda lime with a fresh gas flow rate of 15 L .min^− 1^	3 mg kg^− 1^ propofol, 0.5 g. kg^− 1^ Sufentanil, 0.6 mg kg^− 1^ Rocuronium iv	TIVA with propofol infusion (total dose = 100.6 mg) and bolus doses of sufentanil (total dose = 10 mcg)	invasive blood pressure, ECG, pulse oximetry, nasopharyngeal temperature, end-tidal CO_2_	2 mg kg^− 1^ Tramadol iv
Anaesthetic management of a woman with autosomal recessive limb-girdle muscular dystrophy for emergency caesarean section [20]	T. Allen/2007	A 28-year-old woman	autosomal recessive limb-girdle muscular dystrophy (AR-LGMD)	severe restrictive lung disease	caesarean section	ECG, echocardiogram, Pulmonary function tests	combined spinal-epidural (CSE)	–	–	0.5% hyperbaric bupivacaine 1.8 mL and fentanyl 20 μg injected intrathecally	ECG, pulse oximetry and non-invasive blood pressure	continuous epidural infusion of 0.125% plain bupivacaine, oral paracetamol 1 g Q6h and diclofenac 50 mg Q8h.
Anesthetic management for a child with unknown type of limb-girdle muscular dystrophy [1]	Aysu Kocum/2010	A 4-year-old girl	unknown	–	Adenoidectomy/50 min	–	general anesthesia	–	100 mg propofol, 10 mg Fentanyl iv	6–12 mg.kg^−1^.h^−1^ propofol continuous infusion	ECG, pulse oximetry and non-invasive blood pressure, end-tidal CO2, axillary body temperature	10 mg Meperidine iv
Laparoscopic cholecystectomy under spinal anesthesia in a patient with limb-girdle muscular dystrophy [21]	Michael C. Chuang/2013	A 61-yr-old man	LGMD 2A	dyspneic	laparoscopic cholecystectomy	Echocardiogram, spirometry test	spinal anesthesia	–	–	0.75% hyperbaric bupivacaine 21 mg (2.8 mL) and fentanyl 20 μg. for subarachnoid block, 0.05–0.15 μg.kg^−1^.min^− 1^ remifentanil infusion,	Invasive blood pressure	–
Total intravenous anesthesia for aortic aneurysm replacement surgery in a patient with limb-girdle dystrophy [11]	A. López Álvarez/2013	A 61-year-old male	–	shortness of breath	replacement of ascending aorta /330 min	Echocardiogram	General anesthesia (TIVA)	–	TCI at a target dose 3 - 5 μg/ml for propofol and 2–3 ng/mL for remifentanil, and 1.2 mg/Kg bolus of rocuronium	TCI at a target dose 3 --- 5 μg/ml for propofol and remifentanil 1 --- 3 ng/mL for infusion,	invasive arterial pressure, CVP, 5 lead ECG, pulse oximetry, end-tidal -CO2,BIS, central temperature, NMB, hourly diuresis	fentanyl 300 μg and paracetamol 1 g were administered 30 min before the end of the procedure

Through literature review, we found that respiratory impairment was common in LGMD [10],, and thus the high risk of respiratory compromise should be considered when we administer any anesthesia to a patient with LGMD. Additionally, cardiac impairment should be noticed as well [10]. ECG and Chest X-ray should be performed routinely before surgery, and then for any positive finding, echocardiography and pulmonary function test are suggested. In six previous case reports, two patients underwent laparoscopic cholecystectomy, one patient had appendectomy, one patient underwent caesarean section, one patient had an adenoidectomy, and the last patient went through replacement of ascending aorta. Four operations were performed under TIVA, and the other two operations were performed under neuraxial anesthesia including one laparoscopic cholecystectomy and one caesarean section. Propofol and remifentanil were also adopted as primary anesthetics. If muscle relaxants must be used during operation, neuromuscular blockade monitoring was routinely recommended and sugammadex was used to reverse the blockade at the end of the operation. Among previous case reports, no patient received patient-controlled opioid infusion pumps for postoperative analgesia, and no patient experienced respiratory depression. Our anesthetic strategy was basically in parallel with the regime used in these reports. However, we should keep in mind that the use of TIVA couldn’t completely avoid the risk of MH [22]. Therefore, regional anesthetic techniques should be considered as an alternative plan. Gerbershagen MU et al. suggested regional anesthetic techniques should be chosen whenever possible in myopathic patients [14, 23]. When using regional anesthesia for patients with LGMD, the myotoxic effects of local anesthetic is unfortunately a primary concern. Wolfgang Zink et al. found that continuously infusing bupivacaine and ropivacaine for femoral nerve block could induce acute muscle damage with similar histological patterns [24]. They also found that both agents induced ineversible skeletal muscle damage [25], and the complex pathophysiology of local anesthetic myotoxicity had also been reviewed correspondingly [26]. This phenomenon was also demonstrated by other studies [27–29]. Though many of these studies were experimental in animals, and almost all these studies showed some local muscle damages after continuous peripheral nerve blocks, there were also some case reports in clinic about mytoxicity of local anesthetic [30–32]. In this respect, it might be necessary for a LGMD patient to avoid continuous peripheral nerve block with higher concentration of local anesthetics. In contrast, both epidural and combined spinal-epidural anesthesia have been safely used for LGMD patients receiving cesarean section [1, 20]. So far there was no study about myotoxicity after neuraxial anesthesia. It’s preferred to use neuraxial anesthesia in LGMD patients whenever general anesthesia had to be avoided or carried an increased risk of complication.

In summary, carefully conducted general anesthesia with TIVA technique is a good choice for LGMD patients. Neuraxial anesthesia may be used if general anesthesia needs to be avoided. To warrant safe anesthesia for surgery in LGMD patients, a comprehensive preoperative evaluation, advanced intraoperative monitoring, thoughtful choice of anesthetic technique and pain management strategy, as well as with cooperation of a multidisciplinary team including pulmonologist, critical care physicians, and neuromuscular disease specialists is mandatory.

## Data Availability

The data used or presented during this study are available from the corresponding author on request.

## Abbreviations

EtCO_2_: End-tidal CO_2_
Iv-PCA: Intravenous patient-controlled analgesia
LGMD: Limb-girdle muscular dystrophy
MH: Malignant hyperthermia
TIVA: Total intravenous anesthesia
VAS: Visual analog scale
